# Histopathological changes of acetaminophen-induced liver injury and subsequent liver regeneration in BALB/C and ICR mice

**DOI:** 10.14202/vetworld.2019.1682-1688

**Published:** 2019-11-04

**Authors:** Fazil Muhammad-Azam, Saulol Hamid Nur-Fazila, Raslan Ain-Fatin, Mohamed Mustapha Noordin, Nurhusien Yimer

**Affiliations:** Department of Veterinary Pathology and Microbiology, Faculty of Veterinary Medicine, Universiti Putra Malaysia, 43400 UPM Serdang, Selangor, Malaysia

**Keywords:** acetaminophen, BALB/C, histopathology, institute of cancer research, liver injury, liver regeneration

## Abstract

**Background and Aim:**

Laboratory mice are widely used as a research model to provide insights into toxicological studies of various xenobiotic. Acetaminophen (APAP) is an antipyretic and analgesic drug that is commonly known as paracetamol, an ideal hepatotoxicant to exhibit centrilobular necrosis in laboratory mice to resemble humans. However, assessment of histopathological changes between mouse strains is important to decide the optimal mouse model used in APAP toxicity study. Therefore, we aim to assess the histomorphological features of APAP-induced liver injury (AILI) in BALB/C and Institute of Cancer Research (ICR) mice.

**Materials and Methods:**

Twenty-five ICR mice and 20 BALB/C mice were used where five animals as control and the rest were randomly divided into four time points at 5, 10, 24 and 48 hours post-dosing (hpd). They were induced with 500 mg/kg APAP intraperitoneally. Liver sections were processed for hematoxylin-eosin staining and histopathological changes were scored based on grading methods.

**Results:**

Intense centrilobular damage was observed as early as 5 hpd in BALB/C as compared to ICR mice, which was observed at 10 hpd. The difference of liver injury between ICR and BALB/C mice is due to dissimilarity in the genetic line-up that related to different elimination pathways of APAP toxicity. However, at 24 hpd, the damage was markedly subsided and liver regeneration had taken place for both ICR and BALB/C groups with evidence of mitotic figures. This study showed that normal liver architecture was restored after the clearance of toxic insult.

**Conclusion:**

AILI was exhibited earlier in BALB/C than ICR mice but both underwent liver recovery at later time points.

## Introduction

Acetaminophen (APAP, paracetamol) is a major component in paracetamol that commonly used as antipyretic and analgesic drug in humans. APAP serves as one of the classically studied drugs that characterized as an ideal hepatotoxicant [1]. Metabolism of APAP is carried out by cytochrome P-450 isoform (Cyt P-450) to produce a reactive metabolite known as N-acetyl-p-benzoquinone-imine (NAPQI) [2]. Glutathione (GSH) served as antioxidant and scavenger within hepatocytes which is responsible to detoxify reactive metabolite, reduce oxidative stress, and control redox level [3]. Detoxified NAPQI by GSH will form GSH-NAPQI conjugate to be excreted out as a waste product. However, excessive accumulation of NAPQI produced by APAP overdose depletes cellular GSH that subsequently increases oxidative stress within hepatocytes [4] to exhibit hepatocellular changes. It has been proven that APAP-induced hepatotoxicity in a mouse model is similar to humans as it exhibits mitochondrial damage and nuclear DNA fragmentation, leading to centrilobular necrosis, hemorrhage, and congestion of the liver parenchyma [5]. However, after the clearance of toxic insult at the later time point, subsequent liver regeneration was found to take place after hepatocytes entered the cell cycle [6,7].

Laboratory mice have been extensively used in toxicological studies mainly in APAP toxicity as the mechanism has been proven to resemble humans [8]. Rats possess more resistance against APAP intoxication due to reduction in mitochondrial protein adducts and dysfunction which prevents the production of oxidative stress as compared to mice [9]. Male mice are frequently used due to the fact that the female mice are less susceptible to APAP intoxication due to the low amount of oxidative stress accumulation as a result of rapid recovery of hepatic GSH [10,11]. Meanwhile, different mouse strains can potentially exhibit a different degree of hepatocellular injury [12]. Institute of Cancer Research (ICR) mice are commonly used as a model for toxicological studies, particularly for product safety testing [13]; meanwhile, BALB/C mouse is mainly used for immunological study [14]. ICR mouse is an outbred stock represented wide genetic variability, whereas BALB/C mouse is inbred strain that is considered as genetically homogenous to emit identical response [15]. Although numerous published studies have explored and reported a similar basic mechanism of APAP intoxication in mouse models, hepatotoxic responses for different mouse models are still variable [16]. The comparative studies are yet to be explored to allow the choice of an optimal mouse model to be used in toxicological studies.

This study aimed to assess the histomorphological changes of APAP intoxication in different strains or stock of mice; ICR and BALB/C mice. The histopathological features were described and scored based on the grading system. Moreover, the degree of hepatic regeneration after APAP intoxication was also evaluated.

## Materials and Methods

### Ethical approval

All protocols described were undertaken in accordance with criteria approved by Universiti Putra Malaysia (UPM), Institutional Animal Care and Use Committee (IACUC) – UPM/IACUC/AUP-R078/2017.

### Experimental animals

All animals were purchased from the Animal Resource Unit (ARU), Faculty of Veterinary Medicine, UPM. The experiment was conducted at Animal Research Facility, Faculty of Veterinary Medicine, UPM. The animals had 7 days of acclimatization period before experimentation and they were maintained in a 12 h of light-dark cycle at 7 am and 7 pm with the regulatory temperature at 21-23°C with free access to food and drink.

### Experimental and sampling protocols

A total of 45 male mice (5-6 weeks old, 20-25 g) were used in this study where 5 of them served as controls and another 40 were grouped as APAP-treated animals. In groups of five animals for individual cages, 20 animals for each ICR and BALB/C mice were assigned randomly at four different time points; 5, 10, 24 and 48 h. A tablet of 500 mg/kg APAP was freshly prepared by adding 16.7 mL of 0.9% normal saline at 50 mg/ml concentration into 20 ml glass vial. The APAP solution was dissolved completely before it was administered to individual animals. The dosage of APAP was calculated according to body mass (dose = BWT×16.7 µL) with the range volume of 0.3-0.45 ml per animal. APAP dose of 500 mg/kg is considered as a toxic dose [17] with the exhibition of necrosis mainly on the centrilobular area [10,18]. It is also known to be a sublethal dose that subsequently regenerates the damaged liver cell [16]. The APAP solution was given intraperitoneally at between 8.00 am and 10.00 am. The control animals were administered with 0.9% NaCl intraperitoneally. At four different time frames; 5, 10, 24, and 48 hours post-dosing (hpd), they were humanely euthanized by cervical dislocation. Then, the animals were dissected and liver samples were harvested and fixed in 10% formalin for histological processing.

### Histopathology for the assessment of hepatotoxicity in the liver

Histological specimens from the liver were prepared at the Histopathology Laboratory, Faculty of Veterinary Medicine, UPM. Liver samples were fixed in 10% buffered formalin for at least 24 h before processing. Briefly, the fixed tissues were embedded into the paraffin wax followed by the dehydration process with a series of increasing concentrations of ethanol to remove the free or bound water. The embedded tissues were sliced using a microtome into the tiny section (3-5 µm). For histological assessment, the liver sections were mounted on plain glass slides and routinely stained with Harris’ hematoxylin and eosin (HE) staining method. HE-stained sections were observed for any abnormalities of histopathological features under a light microscope at 100×, 200×, and 400×. The degree of hepatocellular changes was scored based on the grading system done by the previous study [19], as shown in Table-1.

**Table-1:** AILI scores with descriptions on histopathological changes in liver cells that range from 0 (normal hepatocytes) to 5 (severe hepatocytes loss).

Score	Description
0 (−)	Normal–no hepatocytes necrosis
1 (+)	Minimal–mild Focal, limited to centrilobular region Less than ¼ of affected lobules are necrotic
2 (++)	Mild-moderate Focal and multifocal Central to midzonal lobular region ½ affected lobules are necrotic
3 (+++)	Moderate to severe Multifocal (centrilobular-portal region) ¾>*X*>½ affected lobules are necrotic
4 (++++)	Severe Multifocal *X*>¾ affected lobules are necrotic
5 (+++++)	Severe (whole lobules) Hepatocytes loss from central vein to portal area extend to adjacent lobules

AILI=Acetaminophen-induced liver injury

### Statistical analysis

All data obtained were expressed as mean ± standard deviation. Values were analyzed using Shapiro–Wilks test for non-normality data. Normally distributed data were tested using unpaired t-test while Mann–Whitney U-test was used for non-parametric data. All calculations were performed using SPSS statistical software (IBM Corp, NY, USA) and results were considered statistically significant when p<0.05 (*p<0.05, **p<0.01, and ***p<0.005).

## Results

The liver changes observed in 500 mg/kg APAP intoxication toward ICR and BALB/C mice were assessed histologically using HE-stained liver sections. The degree of hepatocellular changes induced by APAP was scored based on the previously applied scoring system by Antoine *et al*. [19]. Descriptions of the histological findings and APAP-induced liver injury (AILI) scores of all animals were done by a pathologist independently and in a blinded fashion. The histopathological features on APAP-induced mice liver were compared at 5, 10, 24, and 48 hpd.

**Figure-1:**
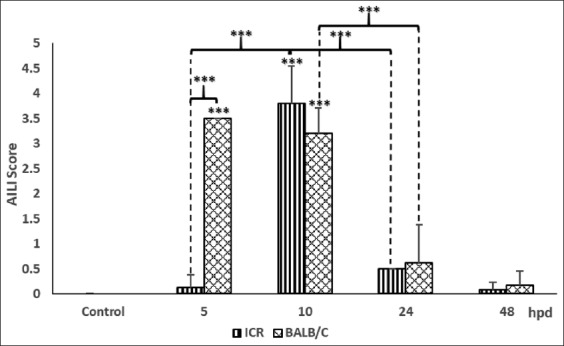
Histopathological grading score of liver samples in controls and 500 mg/kg acetaminophen (APAP)-treated Institute of Cancer Research and BALB/C mice. APAP-induced liver injury scores are determined based on the degree of hepatocellular damage with 0 indicating no histological abnormalities and 5 indicating the most severe damage. Data represent mean±SD (five animals per group). *p<0.05, **p<0.01, and ***p<0.005. hpd: hours post-APAP dosing.

A summary of the average grading scores is recorded in Figure-1. Expectedly, control animals did not reveal any histological abnormalities and they were scored 0. It indicated that no hepatocyte loss and necrosis were observed. Similarly, although both groups of APAP treatment showed hepatocellular injury at all-time points, APAP-treated ICR mice at 5, 24, and 48 hpd showed that no significant difference to controls with AILI score was lesser than 1. Overall, AILI scores for BALB/C groups were higher than ICR groups except at 10 hpd although it differed insignificantly. Intense centrilobular cell damage was significantly observed as early as 5 hpd in BALB/C mice, with individual scoring were ranged between 3 and 4. At 10 hpd, ICR group showed the AILI score of 3.8 while BALB/C mice had continuous hepatocytes injury with a mean score of 3.2, exhibiting the peak of hepatocellular damage could be observed throughout the experimentation. However, the average of AILI scores for BALB/C mice was significantly reduced at 24 and 48 hpd with average score of 0.63 and 0.17, respectively, as demonstrated in Figure-1, with individual animals were scored from 0 to 2.

**Figure-2:**
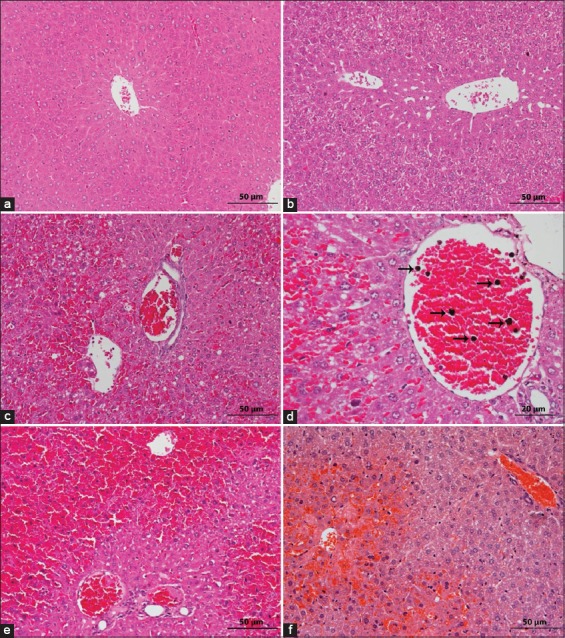
Assessment of histopathological features of liver samples in the Institute of Cancer Research (ICR) and BALB/C mice at 5 and 10 hpd post 500 mg/kg acetaminophen (APAP) treatment (hematoxylin and eosin staining). (a) Normal liver architectures were seen in controls. Scale 50 µm. (b) Mild variable cytoplasmic vacuoles of early hydropic degeneration could be observed in ICR mice at 5 hpd (mean score=0.13). Scale 50 µm. (c) BALB/C mice at 5 hpd exhibited severe hemorrhage with necrosis present on central vein (mean score=3.5) – scale 50 µm, with (d) numerous inflammatory cells (arrows) were observed at 400×. Scale 20 µm. (e) Severe centrilobular necrosis in APAP-treated mice at 10 hpd. Scale 50 µm. (f) Severe hemorrhage and necrosis in the centrilobular region were seen in APAP dose BALB/C mice at the same time frame (mean score=3.2) hpd. Scale 50 µm.

**Figure-3:**
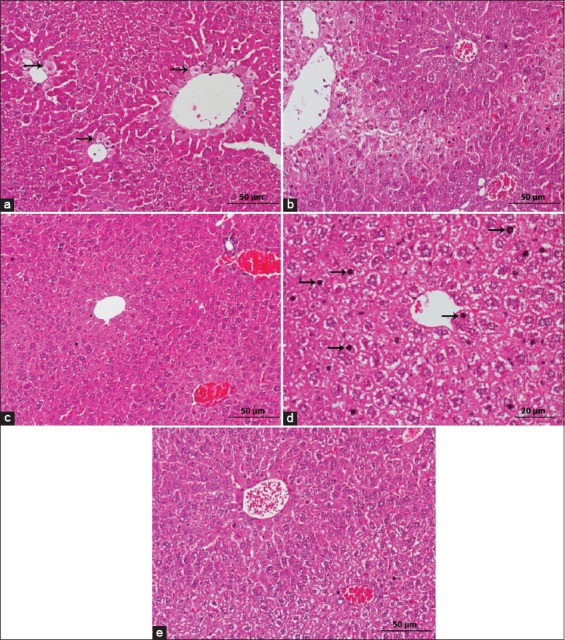
Histomorphological features of liver sections in the Institute of Cancer Research (ICR) and BALB/C mice at 24 and 48 hpd after 500 mg/kg acetaminophen (APAP) induction (hematoxylin and eosin staining). (a) Several hepatocytes swelling (ballooning degeneration) (arrows) surrounded central veins in ICR mice at 24 hpd (mean score=0.5). Scale 50 µm. (b) APAP-treated BALB/C mice at 24 hpd showed persistent mild centrilobular cell damage (mean score=0.63). Scale 50 µm. (c) Complete hepatocytes regeneration in APAP-treated ICR mice at 48 hpd was observed (mean score=0.08) – scale 50 µm; with (d) evidence of mitotic figures (arrows). Scale 20 µm. (e) BALB/C mice revealed the normal architecture of liver section with variable hepatocellular arrangement at the same time point (mean score=0.17) hpd. Scale 50 µm.

Histomorphological features of the liver changes induced by APAP toxicity at 5, 10, 24, and 48 hpd are illustrated in Figures-2-4, respectively. No histological abnormality was recognized in normal saline dose animals, as shown in Figure-2a. Meanwhile, a histological liver section of APAP-treated ICR mice showed variably sized cytoplasmic vacuoles with evidence of early hydropic degeneration and minimal hepatic cell death as early as 5 hpd as observed in Figure-2b. However, at the same time point in APAP-treated BALB/C mice, more severely damaged hepatocytes were observed with evidence of intense centrilobular necrosis and hemorrhage (Figure-2c), with numerous inflammatory cells were seen at 400×, as shown in Figure-2d. At 10 hpd, we observed centrilobular necrosis and hemorrhage that extensively spread to almost 3/4 of liver sections in ICR groups, as shown in Figure-2e. However, BALB/C mice had persistent centrilobular damage (Figure-2f) in which continuously exhibited necrosis as observed in 5 hpd. Both groups revealed great hepatocytes loss and replacement with erythrocytes that coagulated at the hepatocellular regions.

At later time points, hepatic hemorrhage and necrosis were no longer observed and there was replaced by intact hepatocytes. However, minimal existence of centrilobular cellular changes characterized by ballooning degeneration of hepatocytes could be observed in ICR mice at 24 hpd, as illustrated in Figure-3a. Almost complete hepatocellular architectures surrounding the central vein with mild centrilobular necrosis were retained in BALB/C mice (Figure-3b). On the other hand, liver damage had seen in ICR groups was markedly reduced with most of the individual animals showing intact hepatocytes (Figure-3c), with evidence of mitotic figures (Figure-3d) indicative of complete regeneration. Similarly, at 48 hpd, APAP-treated BALB/C mice showed minimal liver cell damage where the normal architecture of hepatic cells was observed in generalized areas of liver section (Figure-3e).

## Discussion

To date, there are several studies that demonstrate the effects of AILI in rodent species to provide an optimal animal model in biomedical research [18,20]. However, histopathological assessment on the strain or stock differences in APAP intoxication is still lacking. Theoretically, different genetic backgrounds in an animal model may influence the outcome of the hepatotoxic responses that should be highlighted to achieve the need for the study. However, the most suitable models used in toxicity studies are still debatable [12,13]. Therefore, a comparative assessment of APAP-induced hepatotoxicity on the liver parenchyma and its subsequent regeneration in outbred ICR and inbred BALB/C mice needs to be further elucidated.

The typical lesion in the liver induced by APAP overdose in mice is well known to exhibit centrilobular necrosis [5,9,19]. Based on AILI grading score to determine the degree of hepatocellular damage following APAP dosing, our study showed that BALB/C mice exhibited extensive liver damage which includes centrilobular cell necrosis, inflammatory cells infiltration, particularly neutrophils, and also ongoing cell death with morphological features of necrosis as compared to ICR mice as early as 5 h of APAP dosing. The previous study showed that different genetic line-up could be the influencing factor for early response dissimilarity [18]. Severe centrilobular hemorrhage and necrosis could be observed in BALB/C than ICR groups possibly due to the susceptibility of BALB/C as inbred strain to acute APAP toxicity. In a study by Fengler *et al*. [21], CD-1 mice as an outbred stock were found to be more resistant in diet-induced liver disease than an inbred strain, C57BL5/J mice with hepatic features of ballooning, inflammation, and lipid accumulation for 7 weeks. Meanwhile, another study also demonstrated similar results, whereby Swiss albino mice had low sensitivity toward toxic shock syndrome toxin as compared to BALB/C mice which showed the highest toxicity [22]. Chen *et al*. [23] mentioned that different elimination pathway of APAP could result in a different response of mice strain induced with APAP intoxication. However, there were no differences in histopathological changes at 10 hpd between ICR and BALB/C where severe centrilobular necrosis was observed. Liver necrosis in APAP intoxication was found to be severe after 6 h post-toxic induction [24] and also observed to be highest at 12 hpd [7].

However, centrilobular damage was substantially reduced and mostly was replaced by intact hepatocytes at the later, 24 and 48 hpd. Both hepatocellular features in ICR and BALB/C mice revealed almost complete regeneration with the presence of numerous mitotic figures at 48 hpd in ICR mice. It had been proven previously that normal liver architecture was restored after clearance of APAP insult in CD-1 mice [25]. The previous studies also provide further evidence that regeneration is the final outcome of AILI in rodent models [6,25]. It was also revealed that liver possesses the ability to self-regenerate after injury induced by APAP toxic insult [19]. In a study conducted by William *et al*. [16], liver necrosis observed in APAP-treated mice was subsided and liver regeneration took place at a later time after clearance of toxic insult due to increase in hepatic GSH to reduce the accumulation of NAPQI and oxidative stress.

Necrotic cells death occurred is the main cause of inflammatory response that may be one of the healing factors at the cellular level. Inflammatory response plays an important role by removing the damaged cells before any cellular mitosis can occur [26]. Two eminent inflammatory cells in AILI are neutrophils and macrophages [27]. Macrophages activation had been known to be beneficial for the proliferation of hepatocytes by removing the damaged cells that are to be replaced with new cells [28]. The presence of mitotic figures at 48 hpd showed that the hepatocytes were regenerated by Bhushan and Apte [29] replacing the damaged cells for the formation of new cells to restore normal liver function. Commonly, cell regeneration is associated with cell mitosis that produces intact cells to replace the damaged cells. In the normal condition of hepatocytes, cells remain quiescence and inactive for cell cycle (G_0_ phase). Induction of AILI, cells start to enter the cell cycle and resume to other phases in mitosis process of G_1_ and S phase where DNA replication occurs followed by G_2_ and mitosis where the cell is divided [30].

Hence, our study proves that different strains and stocks of laboratory mouse could influence the susceptibility of APAP intoxication due to different genetic backgrounds [18], possibly due to the differences in elimination pathways of APAP [23]. Although both showed histopathological lesions at an early time point, both had restored normal hepatocellular architectures and underwent complete hepatic regeneration at later time points.

## Conclusion

The present study demonstrated that different mouse strains respond differently to the same APAP dose. Overall results showed that BALB/C mice had a higher susceptibility of AILI reflected by severe hepatocellular damage as compared to ICR mice at early toxic induction of APAP overdose. Subsequently, complete hepatic regeneration also occurred after clearance of toxic insults at later time frames for both ICR and BALB/C mice. The response exhibited by different mouse strains could not be ignored. Therefore, further investigations would be required to fully understand the mechanism involved to explain the toxicity differences between mouse strains.

## Authors’ Contributions

SHN designed the study, provided the materials and reagents, and performed the histological score and data analysis. FM conducted the experiment, collected the samples, performed statistical analysis, and wrote the manuscript. RA helped with animal study and sample collection. MMN and NY co-supervised the study. SHN and FM contributed to the drafting and revision of the manuscript. All authors read and approved the final manuscript.
